# Intracardiac thrombus in a patient with mitral bioprosthesis and atrial fibrillation treated with direct oral anticoaugulant: A case report

**DOI:** 10.1097/MD.0000000000026137

**Published:** 2021-06-11

**Authors:** Myriam D’Angelo, Roberta Manganaro, Ilaria Boretti, Daniele Giacopelli, Gaetano Cannavà, Francesco Corallo, Placido Bramanti, Antonio Duca

**Affiliations:** a IRCCS Bonino Pulejo of Messina, Cardiology Unit; b Department of Clinical and Experimental Medicine, University of Messina, Cardiology Unit, Azienda Ospedaliera Universitaria “Policlinico G. Martino”, Messina; c Clinical Research, BIOTRONIK Italia, Vimodrone (MI), Italy.

**Keywords:** anticoagulant, atrial fibrillation, mitral bioprosthetic, thrombosis

## Abstract

**Rationale:**

Atrial fibrillation (AF) is the most common cardiac arrhythmia and is associated with increased morbidity, especially stroke and heart failure. There is also increasing awareness that atrial fibrillation is a major cause of embolic events which in 75% of cases are complicated by cerebrovascular accidents.

**Patient concerns:**

A 50-year-old woman with mitral bioprosthesis under warfarin for nonvalvular atrial fibrillation was referred to our Coronary Intensive Care Unit due to acute myocardial infarction without evidence of significant coronary artery stenosis.

**Diagnoses:**

Cardiovascular examination showed an irregular pulse and a grade II diastolic murmur was audible at the apical area. The patient underwent coronary angiography showing absence of obstructive coronary artery disease. We decided to replace Warfarin with direct oral anticoagulants as anticoagulant therapy.

**Interventions:**

Transoesophageal echocardiography revealed a thrombus in left atrial appendage that was treated by replacing warfarin with an oral direct thrombin inhibitor.

**Outcomes:**

At 2-month follow-up, the therapy showed to be effective for thrombus resolution.

**Lessons:**

Our case demonstrated how AF has high risk of thromboembolic complications, not only in terms of stroke but also of myocardial infarction and death.

The use of direct oral anticoagulants in AF patients with bioprosthetic heart valves is still debated due to an unclear definition of “nonvalvular” AF.

## 1 Introduction

Atrial fibrillation (AF) is the most common cardiac arrhythmia^[1]^ and is associated with increased morbidity, especially stroke, and heart failure, as well as increased mortality.^[2]^ Oral anticoagulation with vitamin K antagonists (VKAs) or with direct oral anticoagulants (DOACs) is indicated for AF patients. Different studies compared DOACs with warfarin for prevention of stroke and systemic embolism in patients with nonvalvular AF. DOACs appeared to be safe and effective as a valid alternative to VKAs in patients with nonvalvular AF.^[3–6]^ Current European Guidelines recommend preferring the DOACs over VKAs for stroke prevention in most patients with nonvalvular AF.^[7]^ In patients with bioprosthetic heart valves (BHVs) and AF only VKAs are indicated in the first 3 months postoperatively.^[8]^ After this initial period, there is no general consensus on the alternative use of DOACs due to the lack of prospective controlled studies. We describe the use of DOACs in a young woman with mitral bioprosthesis, initially treated with VKAs (Warfarin) for AF, who developed acute myocardial infarction (AMI) secondary to embolization from left atrial appendage thrombus.

## 2 Case report

We report a case of a 50-year-old Caucasian woman presented with chest pain lasting several hours, associated with profuse sweating. She was admitted to our Coronary Intensive Care Unit with the diagnosis of acute AMI without ST-segment elevation.

She had a history of hypertension, dyslipidemia and diabetes mellitus. In 1988 the patient underwent valvuloplasty for a rheumatic mitral stenosis, while in 2011 the mitral valve was replaced with a biological prosthesis (25 mm Carpentier Edwards). Two months later, after an unsuccessful electrical cardioversion of AF, she had a vertebrobasilar stroke causing rigid-spastic tetraparesis and motor aphasia. From that episode, a strategy of rate control was chosen and anticoagulant therapy with VKAs (Warfarin) was started.

**Figure 1:**
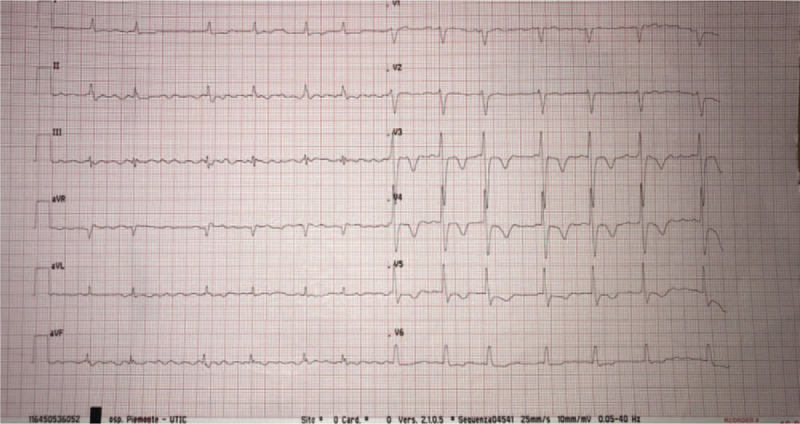
On admission the electrocardiogram showed atrial fibrillation, lateral T wave inversion, and QT interval prolongation.

On admission to our department, physical examination revealed a body temperature of 36.5°C, oxygen saturation of 98% in ambient air, a heart rate of 80 beats/min, a blood pressure of 140/70 mm Hg. Cardiovascular examination showed an irregular pulse and a grade II diastolic murmur was audible at the apical area. Laboratory findings included a white blood cell count of 7300/L (normal range 4000–10,000/L), anemia (Hb 8.5 g/dL), troponin I 13.4 ng/mL, pro-Brain Natriuretic Peptide (NT-pro) 4232 pg/mL, international normalized ratio 2.12. Her CHA2DS2-VASc score was 6 and her HASBLED score was 4. The electrocardiogram confirmed AF, lateral T wave inversion and QT interval prolongation (QTc 0,50 seconds) as shown in Figure 1.

**Figure 2:**
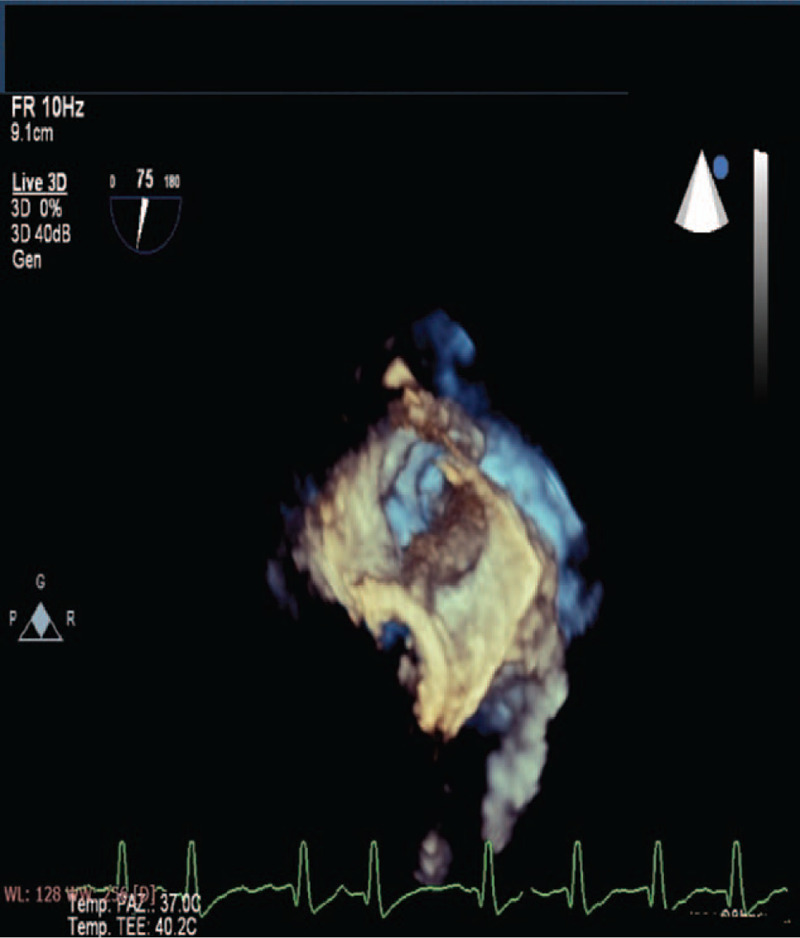
Three-dimensional transesophageal echocardiography showing a left atrial appendage almost entirely occupied by thrombotic material.

Transthoracic echocardiography revealed a severe left ventricle (LV) systolic dysfunction with an ejection fraction of 30%, severe intra-atrial and intraventricular spontaneous echo-contrast effect and slightly increased transprothesic gradients (mean gradient 7 mm Hg at a heart rate of 85–90 beats/min). The patient underwent coronary angiography showing absence of obstructive coronary artery disease. In order to investigate a possible cardioembolic genesis of the AMI and to better evaluate mitral bioprosthesis function, a transesophageal echocardiography was performed. The left atrial appendage (LAA) was almost entirely occupied by thrombotic material, while mitral bioprosthesis was normal in functioning and morphology, as shown in Figure 2.

We decided to replace Warfarin with DOACs (Dabigatran 150 mg twice daily) as anticoagulant therapy. In addition, during the hospitalization the patient developed third degree atrio-ventricular block that led to a dual-chamber implantable cardioverter defibrillator implant, for the concomitant presence of severe LV systolic dysfunction.

At 2 months follow up, we repeated the transesophageal echocardiography that showed resolution of all thrombotic material in the LAA with a mild spontaneous echocontrast effect and persistency of severe LV systolic dysfunction.

## 3 Discussion

Traditionally, VKAs, especially warfarin, have been the cornerstone for stroke prevention in all patients with AF. However, they have a narrow window of therapeutic benefit, a marked variation in their effect in several patients and a need to monitor their action on long-term coagulation. The DOACs overcame these limits and are now recommended in patients with nonvalvular AF. The use of DOAC in patients with AF and bioprostheses beyond 3-month postsurgery, however, is still debated.

Our case demonstrated how AF has high risk of thromboembolic complications, not only in terms of stroke but also of AMI and death. The fear of hemorrhagic complications related to the use of anticoagulant therapy has been and continues to be an obstacle to the correct prescription of this therapy. The main consequence is the underutilization of the therapy with a consequent reduction protection of thromboembolic risk. In addition, an incorrect interpretation of the trials, combined with the exaggerated perception of hemorrhagic risk during treatment with DOAC, has led to the excessive (incorrect and dangerous) use of low doses of the drug. Our patient experienced a thromboembolic event under warfarin. Switching to DOAC showed resolution of thrombus in the LAA.

Although BHVs are less thrombogenic than mechanical heart valves, patients with bioprosthesis and additional risk factors for embolism, such as AF, require life-long therapy with oral anticoagulation. Recently a study suggested that the risk of thromboembolic events in AF patients with bioprosthesis was similar to that of patients with nonvalvular AF without bioprosthesis. Old age and CHA2DS2-VASc score were independent predictors of stroke/systemic thromboembolic events (SEE) and oral anticoagulation was associated with a lower thromboembolic risk. These findings support the concept that patients with AF and BHVs can be assimilated, and therefore treated similarly, to those without significant valvular disease.^[9]^

The use of DOACs in AF patients with BHVs is still debated due to an unclear definition of “nonvalvular” AF. Recently, different new classifications have been proposed. Some authors proposed the term “MARM-AF” to define “Mechanical And Rheumatic Mitral valvular AF.”^[10]^ The functional EHRA (Evaluated Heartvalves, Rheumatic or Artificial) classification relies on the type of oral anticoagulation in patients with AF. The EHRA type 1 includes valvular heart disease that need therapy with only VKAs (moderate-severe mitral stenosis usually of rheumatic origin and mechanical prosthetic valve) while the EHRA type 2 identifies all the other valvular heart diseases in which both VKAs and DOAC can be used (mitral regurgitation, mitral valve repair, aortic stenosis, aortic regurgitation, tricuspid regurgitation, tricuspid stenosis, pulmonary regurgitation, bioprosthetic valve replacements and transaortic valve intervention).^[11]^

According to the European Society of Cardiology Guidelines on AF, patients with AF and BHV or surgical valve repair are eligible to receive DOACs after 3 to 6 months from surgery.^[8]^

The 2018 European Heart Rhythm Association practical guide on the use of DOACs in patients with AF consider DOACs as a valid option in patients with BHVs and AF, except in case of bioprothesis implanted for rheumatic mitral stenosis.^[12]^ In the latter case, in fact, patients usually have atria that remain large and severely diseased, so that VKA may be the preferred option over DOACs. Among the main studies about DOACs, only ARISTOTLE (apixaban for reduction in stroke and other thromboembolic events in atrial fibrillation) and ENGAGE AF (effective anticoagulation with factor Xa next generation in atrial fibrillation) included patients with bioprosthesis or valve repair and AF. In a recent post hoc subgroup analysis they showed that there were no statistically significant interactions (odds) between patients treated with DOACs and those treated with warfarin in terms of stroke, major bleeding or all-cause mortality.

In particular, among 21,105 patients enrolled in the ENGAGE AF (effective anticoagulation with factor Xa next generation in atrial fibrillation), 191 had a previous bioprosthesis valve implantation. There were not significant differences between higher and lower dose of edoxaban vs warfarin in the rate of either stroke or SEE, while both doses of edoxaban were superior to Warfarin in primary net clinical outcome that included stroke/SEE, major bleeding and death.^[13]^

Similar results came from a post hoc analysis of the ARISTOTLE (apixaban for reduction in stroke and other thromboembolic events in atrial fibrillation) that included 104 patients with a bioprosthetic valve (55 were randomized to apixaban and 49 to warfarin). It showed not significant differences between the 2 anticoagulants both in terms of efficacy and safety.^[14]^

The study DAWA (dabigatran vs warfarin after bioprosthesis valve replacement for the management of atrial fibrillation postoperatively) randomized 27 patients: 15 patients received Dabigatran 110 mg twice daily and 12 patients warfarin at least 3 months after bioprosthesis replacement and with AF. The primary endpoint was the evidence of new intracardiac thrombus at 90 days while the secondary endpoint was incidence of myocardium infarction, stroke, valve thrombosis, or dense spontaneous echo contrast. The study was terminated early because of low enrolment rate but did not find significant differences between the 2 groups.^[15]^

A recent retrospective study, conducted on 464 patients with nonvalvular AF and history of bioprosthetic heart valve replacement who received treatment with a DOAC (n = 211) or VKA (n = 253), showed that the use of DOACs was associated with improved net clinical benefit, thanks to a lower incidence of major bleeding and thromboembolic events compared with VKAs.^[16]^

Although larger studies are needed to confirm safety and effectiveness of DOACs in patients with bioprosthesis and AF, their use in these patients is promising as shown in this case report.

## Author contributions

**Conceptualization:** Antonio Duca.

**Investigation:** Myriam D’Angelo.

**Methodology:** Roberta Manganaro, Francesco Corallo.

**Supervision:** Gaetano Cannava, Placido Bramanti.

**Writing – review & editing:** Ilaria Boretti, Daniele Giacopelli.
